# Supplementary material for: Food Choice Motives and COVID-19 in Belgium
Source: Foods. 2022 Mar 15;11(6):842. doi: 10.3390/foods11060842 (PMC8953564; doi:10.3390/foods11060842)
Supplement: Supplementary file 1 [file foods-11-00842-s001.zip › foods-1610266-supplementary.pdf]

**SUPPLEMENTARY MATERIALS S1** Details of the structured online-based survey

The structured online-based survey on a voluntary sample of adult residents from Belgium, as distributed via Microsoft Forms, was available in English, Dutch and French, and covered questions related to participants' characteristics (including socio-demographic information, anthropometric measures and information on COVID-19) as well as the food choice motives applied for the period before (the year 2019) and during (2020-2021) the COVID-19 pandemic (evaluated using the Western Balkan Countries version of the Food Choice Questionnaire (1), originally developed by Steptoe et al. (2)).

**Socio-demographic information**

| Questions in English | Questions in Dutch | Questions in French |
|---|---|---|
| Gender<br>- Male<br>- Female<br>- X, prefer not to say | Geslacht<br>- Man<br>- Vrouw<br>- X, zeg ik liever niet | Sexe<br>- Homme<br>- Femme<br>- X, je préfère ne pas répondre |
| Age<br>- Number of years | Leeftijd<br>- … jaren | Âge<br>- … ans |
| What is your highest level of education completed?<br>- A few grades of primary school<br>- Primary school<br>- Secondary school<br>- Bachelor's degree<br>- Master's degree<br>- Doctorate degree | Welk is het hoogste diploma of de hoogste graad die u behaald heeft?<br>- Geen diploma<br>- Lager onderwijs<br>- Secondair onderwijs<br>- Bachelor diploma<br>- Master diploma<br>- Doctoraat | Quel est le diplôme ou le degré le plus élevé que vous ayez obtenu jusqu' à présent ?<br>- Pas de diplôme ou enseignement primaire<br>- Enseignement secondaire<br>- Grade de bachelier<br>- Grade de master<br>- Grade de docteur |
| What is current employment status?<br>- Employed<br>- Unemployed<br>- Retired<br>- Student | What is uw huidige tewerkstelling?<br>- Betaald werk<br>- Werkloos/ziekte<br>- Onbetaald huishoudelijk werk<br>- Gepensioneerd<br>- Student | Quelle est votre situation professionnelle actuelle?<br>- Employé<br>- Sans emploie<br>- Retraité<br>- Etudiant |
| If you are currently employed, do you work in the healthcare system or is your job directly related to the healthcare system?<br>- Yes<br>- No | Indien u momenteel betaald word verricht, heeft u werk in de gezondheidszorg of werk direct gerelateerd aan het gezondheidssysteem?<br>- Ja<br>- Nee | Si vous êtes actuellement employé(e), travaillez-vous dans le système de santé ou votre emploi est-il directement lié au système de santé ?<br>- Oui<br>- Non |
| What is your marital status<br>- Unmarried<br>- Married/cohabitation<br>- Divorces/separated<br>- Widowed | Wat is uw burgerlijke status?<br>- Ongehuwd/alleenstaand<br>- Gehuwd/samenwonend<br>- Gescheiden<br>- Weduwnaar/ weduwe | Quel est votre état civil?<br>- Vivant seul<br>- Marié(e)/cohabitation<br>- Divorcé(e)/séparé(e)<br>- Veuf/veuve |
| Where is your current residence located? | Hoe omschrijft u uw woonomgeving? | Ou se trouve votre résidence actuelle? |

| | | |
|---|---|---|
| - Urban area (city)<br>- Rural area (countryside) | - Stedelijk gebied (stad)<br>- Landelijk gebied (platteland) | - Zone urbaine (ville)<br>- Zone rurale (campagne) |
| What is your average monthly net income?<br>- I have no independent income.<br>- Less than 1500 EUR<br>- 1500-3550 EUR<br>- More than 3550 EUR | Wat is uw gemiddeld maandelijks inkomen (netto)?<br>- Inkomen als zelfstandige<br>- Minder dan 1500 EUR<br>- 1500-3550 EUR<br>- Meer dan 3550 EUR | Quel est votre revenu mensuel moyen net?<br>- Je n'ai pas de revenu indépendant<br>- Moins de 1600EUR<br>- 1600-2000EUR<br>- Plus de 3550EUR |

**Anthropometrics**

| Questions in English | Questions in Dutch | Questions in French |
|---|---|---|
| Body weight<br>- … kg | Lichaamsgewicht<br>- … kg | Poids corporel<br>- … kg |
| Body height<br>- … cm | Lichaamslengte<br>- … cm | Taille corporelle<br>- …cm |

**Information on COVID-19**

| Questions in English | Questions in Dutch | Questions in French |
|---|---|---|
| Were you infected with COVID-19?<br>- Yes<br>- No | Bent u geïnfecteerd geweest met COVID-19?<br>- Ja<br>- Nee | Avez-vous été infecté(e) par le COVID-19 ?<br>- Oui<br>- Non |
| If you were tested positive for COVID-19, how was the infection detected?<br>- By PCR test<br>- By rapid antigen test<br>- By serology test<br>- The virus infection was not confirmed by any of the above-mentioned methods | Indien u positief testte voor COVID-19, hoe werd de infectie vastgesteld?<br>- Via een PCR test<br>- Via een snelle antigeen test<br>- Via een serologische test<br>- De virusinfectie werd niet bevestigd door één van de bovengenoemde testmethodes. | Si vous avez été testé(e) positif au COVID-19, comment l'infection a-t-elle été détectée?<br>- Par un test PCR<br>- Par un test antigénique rapide<br>- Par un test sérologique<br>- L'infection virale n'a été confirmée par aucune des méthodes mentionnées ci-dessus. |
| Have you been vaccinated against COVID-19?<br>- Yes, fully vaccinated<br>- Yes, partly vaccinated<br>- No | Bent u reeds gevaccineerd tegen COVID-19?<br>- Ja volledig<br>- Ja gedeeltelijk<br>- Nee | Avez-vous été vacciné(e) contre le COVID-19 ?<br>- Oui, entièrement<br>- Oui, partiellement<br>- Non |
| Did you ever have to self-isolate due to COVID-19 preventive measures?<br>- Yes<br>- No | Heeft u zichzelf moeten isoleren omwille van COVID-19 preventiemaatregelen?<br>- Ja<br>- Nee | Avez-vous déjà dû vous auto-isoler en raison des mesures de prévention contre le COVID-19?<br>- Oui<br>- Non |
| Was anyone from your household infected with COVID-19?<br>- Yes<br>- No | Heeft iemand in uw huishouden een COVID-19 infectie doorgemaakt?<br>- Ja<br>- Nee | Quelqu'un de votre ménage a-t-il été infecté par le COVID-19 ?<br>- Oui<br>- Non |

## Food Choice Questionnaire items

| Questions in English | Questions in Dutch | Questions in French |
|---|---|---|
| Please rate the extent to which you agree or disagree with the following statements. The listed statements aim to assess your general standings towards food BEFORE the COVID-19 pandemic. Some statements are very similar to each other, but we kindly ask you to answer all of them because each one refers to some special characteristic related to your eating habits. Please use a 5-point scale, in which 1 means that you strongly disagree, 2 disagree, 3 neither agree nor disagree, 4 agree, and 5 that you strongly agree. | Gelieve aan te geven in hoeverre u het eens of oneens bent met de volgende stellingen. Onderstaande stellingen zijn bedoeld om uw algemeen standpunt ten aanzien van voeding VOOR de COVID-19 pandemie te kunnen beoordelen. Sommige stellingen lijken op elkaar, maar we willen u vriendelijk vragen om ze allemaal te beantwoorden omdat ze elks verwijzen naar een bepaald kenmerk gerelateerd aan uw eetgewoontes. Gelieve de stellingen te beoordelen met een 5-puntenschaal waarbij 1 betekent dat u het er helemaal oneens mee bent en 5 helemaal eens. | Veuillez indiquer dans quelle mesure vous êtes d'accord ou non avec les affirmations suivantes. Les affirmations ont pour but d'évaluer votre position générale en matière d'alimentation AVANT la pandémie de COVID-19. Certaines affirmations sont très similaires les unes aux autres, mais nous vous demandons de bien vouloir répondre à toutes, car chacune d'entre elles fait référence à une caractéristique particulière liée à vos habitudes alimentaires. Veuillez utiliser une échelle de 5 points, dans laquelle 1 signifie que vous n'êtes pas du tout d'accord et 5 que vous êtes tout à fait d'accord. |
| The same food choice items were asked for the period DURING the COVID-19 pandemic. | Dezelfde voedingskeuze-items zijn bevraagd voor de periode TIJDENS de pandemie. | Les mêmes choix alimentaires ont été demandés pour la période PENDANT la pandémie de COVID-19. |

| | Items in English | Items in Dutch | Items in French |
|---|---|---|---|
| Instructions | It is important to me that the food I eat on a typical day: | Voor mij is het belangrijk dat wat ik eet op een typisch dag: | It est important pour moi ce que je mange au cours d'une journée typique : |
| Health | keeps me healthy | Me gezond houdt | Me garde en bonne santé |
| | is nutritious | Een hoge voedingswaarde heeft | Est nutritive |
| | is high in protein | Rijk aan eiwitten is | Soit riche en protéines |
| | is high in fibre and roughage | Rijk is aan vezels | Est riche en fibres et roughage |
| | contains lots of vitamins and minerals | Veel vitamines en mineralen bevat | Contient beaucoup de vitamines et de minéraux |
| | is good for my skin/teeth/hair/nails etc | Goed voor mijn huid/tanden/haar/nagels enz. is | Est bonne pour ma peau/mes dents/mes cheveux/mes ongles, etc. |
| Convenience | is easy to prepare | Makkelijk te bereiden is | Soit facile à préparer |
| | can be cooked very simply | Makkelijk te koken is | Soit facile à cuisiner |
| | takes no time to prepare | Geen tijd vraagt om te bereiden | Ne prend pas de temps à préparer |
| | is easily available in shops and supermarkets | Gemakkelijk beschikbaar is in winkels/supermarkten | Est facilement disponible dans les magasins et les supermarchés |
| | can be bought in shops close to where I live or work | Kan worden gekocht in winkels dichtbij waar ik woon of werk | Peut être achetée dans les magasins proches de ma maison ou de mon travail |
| Sensory appeal | tastes good | Goed smaakt | A bon goût |
| | looks nice | Er goed uitziet | A l'air bien |

| | | | |
|---|---|---|---|
| | smells nice | Goed ruikt | Sent bon |
| | has a pleasant texture | Een aangename textuur heeft | A une texture agréable |
| Natural content | contains natural ingredients | Enkel natuurlijke ingrediënten bevat | Contient des ingrédients naturels |
| | contains no artificial ingredients | Geen kunstmatige ingrediënten bevat | Ne contient aucun ingrédient artificiel |
| | contains no additives | Geen additieven bevat | Ne contient aucun additif |
| Ethical concern | is packaged in an environmentally friendly way | Verpakt is op een milieuvriendelijke manier | Est emballée de manière écologique |
| | has the country of origin clearly marked | Het land van herkomst duidelijk heeft aangegeven | A le pays d'origine clairement indiqué |
| | comes from countries I approve of politically | Afkomstig is van landen die ik politiek goedkeur | Provient de pays que j'approuve politiquement |
| Weight control | is low in calories | Laag in calorieën is | Est faible en calories |
| | is low in fat | Laag in vet is (weinig vet bevat) | Est pauvres en matière gras |
| | helps me control my weight | Mij helpt om mijn gewicht onder controle te houden | M'aide à contrôler mon poids |
| Mood | cheers me up | Mij opvrolijkt | Me réconforte |
| | makes me feel good | Mij goed doet voelen | Me fait me sentir bien |
| | keeps me awake and alert | Me wakker en alert houdt | Me garde éveillé et alerte |
| | helps me cope with stress | Mij helpt om met stress om te gaan | M'aide à gérer le stress |
| | helps me relax | Mij helpt om te relaxen | M'aide à me relaxer |
| | helps me to cope with life | Mij helpt om met mijn leven om te gaan | M'aide à faire face à la vie |
| Familiarity | is familiar to me | Bekend is voor mij | M'est familière |
| | is what I usually eat | Is wat ik gewoonlijk eet | Est-ce que je mange habituellement |
| | is like the food I ate when I was a child | Is zoals de voeding die ik at toen ik kind was | Soit comme la nourriture que j'ai mangée quand j'étais enfant |
| Price | is not expensive | Niet duur is | N'est pas chère |
| | is cheap | Goedkoop is | Est bon marché |
| | is good value for money | Een goede prijs/kwaliteit verhouding heeft | Est d'un bon rapport qualité-prix |

## References


1. Milošević J, Žeželj I, Gorton M, Barjolle D. Understanding the motives for food choice in Western Balkan Countries. Appetite. 2012;58:205-214.
2. Steptoe A, Pollard TM, Wardle J. Development of a measure of the motives underlying the selection of food: the food choice questionnaire. Appetite. 1995;25:267-284.
